# Posterior white matter integrity and self-reported posterior cortical symptoms using the Colorado Posterior Cortical Questionnaire

**DOI:** 10.3389/fneur.2023.1072938

**Published:** 2023-02-01

**Authors:** Samantha K. Holden, Brianne M. Bettcher, Christopher M. Filley, Dan Lopez-Paniagua, Victoria S. Pelak

**Affiliations:** ^1^Department of Neurology, University of Colorado School of Medicine, Aurora, CO, United States; ^2^Department of Neurology, Behavioral Neurology Section, University of Colorado School of Medicine, Aurora, CO, United States; ^3^Department of Neurology, Movement Disorders Section, University of Colorado School of Medicine, Aurora, CO, United States; ^4^Department of Psychiatry, University of Colorado School of Medicine, Aurora, CO, United States; ^5^Marcus Institute for Brain Health, Aurora, CO, United States; ^6^Department of Radiology, University of Colorado School of Medicine, Aurora, CO, United States; ^7^Department of Ophthalmology, University of Colorado School of Medicine, Aurora, CO, United States

**Keywords:** white matter, posterior cortex, outcome measure assessment, posterior cortical atrophy (PCA), self-report measures, diffusion tensor imaging

## Abstract

**Background:**

The Colorado Posterior Cortical Questionnaire (CPC-Q) is a self-report, 15-item screening questionnaire for posterior cortical symptoms, including visuospatial and visuoperceptual difficulties. Changes in white matter connectivity may precede obvious gray matter atrophy in neurodegenerative conditions, especially posterior cortical atrophy. Integration of CPC-Q scores and measures of white matter integrity could contribute to earlier detection of posterior cortical syndromes.

**Methods:**

We investigated the relationships between posterior cortical symptoms as captured by the CPC-Q and diffusion tensor imaging fractional anisotropy (DTI FA) of white matter regions of interest localized to posterior brain regions (posterior thalamic radiations, splenium of corpus callosum, tapetum). Comparisons were also made by diagnostic group [healthy older adult (*n* = 31), amnestic Alzheimer's disease (AD, *n* = 18), and posterior cortical atrophy (PCA, *n* = 9)] and by SENAS battery visuospatial composite score quartile. Exploratory comparisons of all available individual white matter region DTI FA to CPC-Q, as well as comparisons of DTI FA between diagnostic groups and visuospatial quartiles, were also made.

**Results:**

CPC-Q score was correlated with the average DTI FA for the averaged posterior white matter regions of interest (r = −0.31, *p* = 0.02). Posterior thalamic radiation DTI FA was most strongly associated with CPC-Q (r = −0.34, *p* = 0.01) and visuospatial composite (r = 0.58, *p* < 0.01) scores and differed between the PCA and AD groups and the lower and higher visuospatial quartiles. The DTI FA of body and splenium of the corpus callosum also demonstrated this pattern but not the DTI FA of the tapetum.

**Conclusion:**

The integrity of posterior white matter tracts is associated with scores on the CPC-Q, adding to the validation evidence for this new questionnaire. White matter regions that may be related to posterior cortical symptoms detected by the CPC-Q, and distinct from those affected in amnestic syndromes, include the posterior thalamic radiations and body and splenium of the corpus callosum. These findings are in line with previous neuroimaging studies of PCA and support continued research on white matter in posterior cortical dysfunction.

## Introduction

Posterior cortical symptoms, including visuospatial and visuoperceptual impairments, are under-recognized by both clinicians and patients (1). Patients with these symptoms may first present to ophthalmology or optometry clinics for perceived eye problems; alternatively, they may seek neurological evaluation for perceived memory problems. Solicitation of qualitative description of these problems from patients uncovers non-ocular, non-amnestic symptoms. In addition to visually-based deficits, posterior cortical dysfunction can also cause difficulty with calculations, left/right orientation, and praxis (2). Posterior cortical symptoms are often more disabling and anxiety-provoking than other types of cognitive symptoms, with more prominent and earlier safety issues (e.g., driving impairment, falls) (3, 4). Systematic screening for posterior cortical symptoms could improve accuracy and timeliness of diagnoses, leading to improved symptom management, counseling, and outcomes. To contribute to this goal, our group recently developed a brief self-report posterior cortical symptom screening tool, the Colorado Posterior Cortical Questionnaire (CPC-Q) (5). The CPC-Q demonstrates strong psychometric properties, correlates strongly with visuospatial neuropsychological measures, and can distinguish participants with expert consensus posterior cortical atrophy (PCA) diagnosis from participants who are asymptomatic or have amnestic Alzheimer's disease (AD).

To extend this prior work and further validate the CPC-Q, we assessed the neuroanatomical correlates of the measure with a specific focus on white matter integrity. Visuospatial and visuoperceptual symptoms are not restricted to dysfunction of posterior cortical regions. More widespread structural and functional networks also play a major role, including fronto-parietal and temporal-parietal regions. Connectivity disruption may be more sensitive for higher order visual dysfunction than regional atrophy patterns, especially at the earliest stages of neurodegenerative conditions (6). Changes in white matter tracts have been demonstrated in several diffusion tensor imaging (DTI) studies of PCA, with these changes exceeding what would be expected based on gray matter atrophy of posterior regions alone (7–10). One study found significantly more white matter degeneration in PCA than in logopenic primary progressive aphasia, another “atypical” form of AD, suggesting that white matter may be particularly vulnerable in PCA (10). Specific white matter tracts implicated in PCA by these imaging studies include the posterior thalamic radiations, splenium of the corpus callosum, inferior longitudinal fasciculus, and the cingulum.

For our goal of early detection of posterior cortical syndromes, including but not limited to PCA, we included a broader group of participants with a range of quality and severity of cognitive symptoms. We aimed to explore the relationship between the CPC-Q and structural white matter alterations associated with posterior cortical dysfunction. The clinical utility of this screening questionnaire would improve if it were also associated with subtle and early white matter changes, even prior to obvious cortical atrophy. Informed by previous work focused on PCA, our primary hypothesis was that total CPC-Q scores would negatively correlate with the fractional anisotropy (FA) of white matter tracts localized to posterior brain regions (i.e., greater posterior cortical symptom burden, reflected by higher CPC-Q scores, are associated with lower integrity posterior white matter tracts), namely the posterior thalamic radiations, the splenium of the corpus callosum, and the tapetum. However, because posterior cortical symptoms involve broader structural connections with the temporal and frontal cortices, we also examined additional white matter tracts in exploratory analyses to determine if more widespread white matter alterations were associated with self-reported posterior cortical symptoms on the CPC-Q.

## Materials and methods

### Participants and clinical assessment

This cross-sectional study took place within an ongoing longitudinal, observational study of cognitive aging at the University of Colorado Alzheimer's and Cognition Center, the Longitudinal Biomarker and Clinical Phenotyping (Bio-AD) study. Inclusion and exclusion criteria for the Bio-AD study, as well as methods for cognitive and syndromic classification by consensus conference have been described previously (5). Fifty-nine Bio-AD participants completed the CPC-Q, along with clinical interview, neurological examination, cognitive testing [Montreal Cognitive Assessment (MoCA) (11) and Spanish and English Neuropsychological Assessment Scales (SENAS) (12)]. Informant-based interviews were performed for determination of presence and severity of functional impairment [Clinical Dementia Rating (CDR) (13)]. Cognitive domain (visuospatial, executive, memory, and language) composite scores were calculated from the SENAS battery item response theory (IRT) composite scores. Administration procedures, measure development, and psychometric characteristics of the SENAS battery are described in detail elsewhere (14). All participants provided written informed consent and the study protocol was approved by the Colorado Multiple Institutional Review Board (Protocol #15-1774).

### MRI acquisition and neuroimaging analysis

Whole brain MRI scans were obtained on a 3.0 Tesla Siemens (Iselin, NJ) Skyra scanner equipped with a 20- channel head coil. Diffusion imaging data were acquired utilizing multi-shell 2D echo planar imaging sequence (56 slices; TR/TE = 8,400/105 milliseconds, acquisition matrix = 112 × 112; 2 × 2 × 2 mm^3^ isotropic spatial resolution; 64 x b = 2,500 s/mm^2^ A>P direction; 32 x b = 700 s/mm^2^ P>A direction; 18 x b = 0 s/mm^2^). Diffusion data were first pre-processed to correct for susceptibility induced distortions using FMIRB Software Library (FSL) tools *topup* function, as well as eddy current distortions and motion artifacts using FSL's *eddy* function (15, 16). The corrected diffusion data were imported into DSI studio (https://dsi-studio.labsolver.org to create the diffusion maps. Region of Interest (ROI) analyses were performed by first registering individual subject's FA maps to the MNI152_T1_1mm template using an affine transformation in DSI studio. Mean FA values were then extracted from 48 white matter regions (including left and right hemisphere regions separately) obtained from the Johns Hopkins University ICBM-DTI-81 atlas (17).

**Figure 1:**
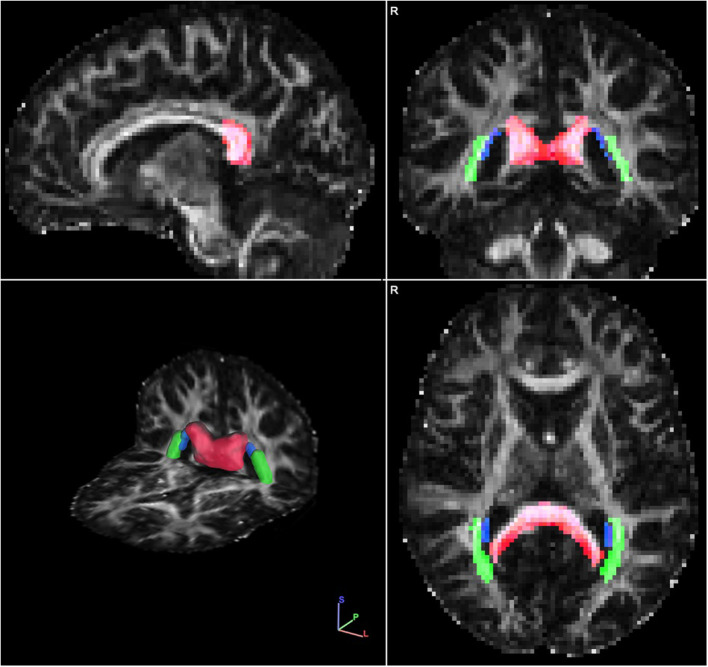
Single representative diffusion fractional anisotropy (FA) map with the *localized posterior tract* ROIs overlayed in sagittal **(Upper left quadrant)**, coronal **(Upper right quadrant)**, axial **(Bottom right quadrant)** and 3-dimensional **(Bottom left quadrant)** views. The ROIs include: splenium of the corpus callosum (red), tapetum (blue), and posterior thalamic radiations (green). Images are displayed in radiological view.

Posterior white matter tracts of interest included the posterior thalamic radiations, splenium of the corpus callosum, and tapetum, with the justification that these tracts are localized only to posterior brain regions without more diffuse projections and would therefore be expected to be strongly associated with clinical symptoms captured by the CPC-Q (Figure 1). FA measurement for these three tracts were averaged with an equal weighting to create a *localized posterior tracts* FA variable, allowing for greater variability.

### Statistical analyses

Demographic and clinical characteristics of participants were evaluated using descriptive statistics. To evaluate the white matter integrity of the full cohort by objectively measured visuospatial function, independent of diagnostic group (healthy older adult, amnestic AD, or PCA), the cohort was divided into quartiles based on visuospatial composite score from the SENAS battery. Mean DTI FA for all available white matter regions/tracts was calculated and compared for the resulting four groups, with Quartile 1 representing the lowest visuospatial composite scores and Quartile 4 representing the highest. Mean DTI FA for all available white matter regions/tracts was also compared by diagnostic group. Normality of CPC-Q and DTI FA data was tested using Shapiro-Wilk W tests; based on these results, one-way ANOVA or Kruskal-Wallis one-way ANOVA analyses were then performed, as appropriate, to evaluate for groupwise differences in DTI FA for each white matter region/tract. Groupwise differences were then tested by either *t*-tests or Mann Whitney U tests, as appropriate.

The association between the CPC-Q and FA of the *localized posterior tracts* was tested with pairwise correlation. A linear regression model, with CPC-Q total score as the dependent variable, was also used to evaluate the relationship with the *localized posterior tract* FA variable, with relevant covariates of participant age and education. As exploratory analysis into the relationship between the CPC-Q and integrity of white matter, pairwise correlations (with Bonferroni correction for multiple comparisons) were also calculated for all available regions from our dataset, including those tracts that should not be associated with posterior cortical function (e.g., corticospinal tracts). For comparison, pairwise correlations were also calculated for all ROIs and visuospatial composite scores, evaluating the hypothesis that the strength of the relationship between CPC-Q score and posterior white matter tracts should mirror that of the relationship between objectively measured visuospatial function and posterior white matter tracts. Statistical analyses were performed using the Stata statistical software package (StataCorp 2015. Stata Statistical Software: Release 14. College Station, TX: StataCorp LP).

## Results

### Participant characteristics

Demographic and clinical characteristics of the participants are presented in Table 1. There was a broad range of age (54–83 years), education (12–20 years), and global cognitive abilities [Montreal Cognitive Assessment (MoCA) (11) score range 7–30] among participants. Of the 59 participants, 31 (53%) were healthy older adults, 19 (32%) had amnestic AD, and 9 (15%) had PCA. Scores on the CPC-Q ranged from 0 to 44, with a mean score of 7 and median score of 4, with a positive skew of data distribution. The PCA group was significantly younger (mean diff 9.2 years, *p* < 0.01) with significantly longer duration of symptoms (mean diff 1.6 years, *p* = 0.04) compared to the amnestic AD group. Visuospatial composite scores ranged from −2.9 to 0.03 for Quartile 1 (*n* = 17, mean CPC-Q score 14.2 ± 15.4); 0.03–0.45 for Quartile 2 (*n* = 12, mean CPC-Q score 3.1 ± 3.4); 0.45–0.86 for Quartile 3 (*n* = 12, mean CPC-Q score 3.1 ± 3.4); and 0.86–2.1 for Quartile 4 (*n* = 18, mean CPC-Q score 3.7 ± 4.0).

**Table 1:** Demographic and clinical characteristics.

	**Total cohort (*n* = 59)**	**Healthy older adult (*n* = 31)**	**Amnestic AD (*n* = 19)**	**PCA (*n* = 9)**
Age (y)	70.5 ± 7.0 (54–83)	69.1 ± 6.5 (55–82)	75 ± 4.6 (63–83)	65.8 ± 8.6 (54–78)
Gender (%F)	59%	71%	42%	56%
Education (y)	17 ± 2.1 (12–20)	17 ± 1.8 (13–20)	17.1 ± 2.3 (13–20)	16.7 ± 3 (12–20)
Symptom duration (y)			3.7 ± 1.9 (2–8)	5.3 ± 1.7 (3–9)
MoCA	23 ± 5 (7–30)	26 ± 2 (23–30)	21± 3 (15–27)	16 ± 4 (7–21)
CDR	0: 53% 0.5: 28% 1: 10% 2: 9%	0: 100% 0.5: 0% 1: 0% 2: 0%	0: 0% 0.5: 72% 1: 17% 2: 11%	0: 0% 0.5: 33% 1: 33% 2: 33%
Visuospatial composite score	0.1 ± 1.2 (-2.9–2.1)	0.7 ± 0.5 (-0.4–2.1)	0.3 ± 0.6 (-1.0–1.1)	−2.3 ± 0.7 (−2.9– −1.2)
Verbal memory composite score	0.4 ± 1.0 (−1.8–2.0)	1.1 ± 0.6 (−0.2–2.0)	−0.4 ± 0.7 (−1.8–1.3)	−0.5 ± 0.9 (−1.6–1.5)
Executive composite score	0.2 ± 0.6 (−1.8–1.3)	0.6 ± 0.4 (−0.3–1.3)	−0.1 ± 0.4 (−1.3–0.6)	−0.5 ± 0.7 (−1.8–0.7)
Language composite score	0.3 ± 0.6 (−1.3–1.9)	0.6 ± 0.6 (−0.8–1.9)	0.1 ± 0.6 (−1.3–1.1)	0.02 ± 0.6 (−0.7–1.0)
Total CPC-Q score	7 ± 10 (0–44)	3 ± 4 (0–14)	5 ± 5 (0–14)	24 ± 15 (7–44)

Data presented as mean ± SD (range) unless otherwise specified. AD, Alzheimer's disease; PCA, Posterior cortical atrophy; MoCA, Montreal Cognitive Assessment; CDR, Clinical Dementia Rating; CPC-Q, Colorado Posterior Cortical Questionnaire.

### White matter integrity by diagnostic group and visuospatial quartile

Mean DTI FA measurements of all available white matter region/tracts are presented in Table 2, for the entire cohort and by diagnostic groups. Several white matter tracts demonstrated significant differences in FA between the healthy older adult and either amnestic AD or PCA groups, with lower FA in the symptomatic groups. Only three tracts were significantly different between the two symptomatic groups, with lower FA in the PCA group for the body of the corpus callosum, posterior thalamic radiations, and splenium of the corpus callosum when compared to the amnestic AD group.

**Table 2:** Mean DTI FA for all white matter region/tracts by diagnostic group.

**White matter region/tract**	**DTI FA**			
	**Entire cohort** **(*****n*** = **59)**	**Healthy older adult** **(*****n*** = **31)**	**Amnestic AD** **(*****n*** = **19)**	**PCA** **(*****n*** = **9)**
**Anterior corona radiata**	0.363	**0.373** ^*+^	**0.353** *^**+**^*	**0.345** ^*^
Anterior limb of internal capsule	0.467	0.474	0.463	0.451
***Body of corpus callosum***	0.517	**0.531** ^*^	**0.511** ^**#**^	**0.479** ^*#^
**Cerebral peduncle**	0.529	**0.538** ^*^	0.526	**0.508** ^*^
**Cingulum**	0.336	**0.354** ^*^	0.330	**0.291** ^*^
Corticospinal tract	0.530	0.534	0.525	0.526
External capsule	0.398	0.403	0.397	0.384
**Fornix column body**	0.282	**0.339** ^*+^	**0.211** *^+^*	**0.236** ^*^
**Fornix cres**	0.413	**0.438** ^*+^	**0.396** *^+^*	**0.362** ^*^
**Genu of corpus callosum**	0.450	**0.477** ^*+^	**0.424** *^**+**^*	**0.416** ^*^
**Hippocampal white matter**	0.315	**0.332** ^*+^	**0.300** *^**+**^*	**0.284** ^*^
Inferior cerebellar peduncle	0.479	0.482	0.480	0.467
Medial lemniscus	0.532	0.529	0.540	0.521
Middle cerebellar peduncle	0.486	0.486	0.491	0.479
Pontine crossing tract	0.444	0.451	0.442	0.422
Posterior corona radiata	0.426	0.433	0.424	0.408
**Posterior limb of internal capsule**	0.527	**0.533** ^*^	0.525	**0.513** ^*^
***Posterior thalamic radiations***	0.472	**0.485** ^*^	**0.467** ^**#**^	**0.435** ^*#^
Retrolenticular internal capsule	0.539	0.536	0.545	0.533
**Sagittal stratum**	0.431	**0.439** ^*^	0.427	**0.413** ^*^
***Splenium of corpus callosum***	0.558	**0.574** ^*^	**0.551** ^**#**^	**0.517** ^*#^
Superior cerebellar peduncle	0.575	0.575	0.585	0.553
Superior corona radiata	0.435	0.431	0.443	0.429
**Superior fronto-occipital fasciculus**	0.390	**0.407** ^**+**^	**0.369** ^**+**^	0.377
Superior longitudinal fasciculus	0.440	0.444	0.438	0.430
**Tapetum**	0.340	**0.368** ^+*^	**0.310** ^**+**^	**0.303** ^*^
**Uncinate fasciculus**	0.408	**0.41** ^*^	0.406	**0.378** ^*^
**Localized posterior tracts**	0.456	**0.476** ^+*^	**0.443** ^**+**^	**0.418** ^*^

^*^Significant difference between healthy older adults and PCA; ^+^significant difference between healthy older adults and amnestic AD; ^#^significant difference between amnestic AD and PCA, correcting for multiple comparisons (Bonferroni). **Bold** indicates differences between asymptomatic and symptomatic groups. **Bold italic** indicates differences between symptomatic syndromes: amnestic AD vs. PCA.

Table 3 displays mean white matter region/tract FA by visuospatial composite quartiles and statistical differences between groups. Ten tracts demonstrated significant groupwise differences between the lowest (Quartile 1) and highest (Quartile 4) visuospatial composite score groups. The FA of five tracts were significantly different between Quartile 1 and Quartile 3, but only two tracts were significantly different between Quartile 1 and 2 (fornix cres and hippocampal white matter).

**Table 3:** Mean DTI FA for all white matter region/tracts by visuospatial composite quartile.

**White matter region/tract**	**DTI FA**			
	**Quartile 1** ***(lowest vs. performance)***	**Quartile 2**	**Quartile 3**	**Quartile 4** ***(highest vs. performance)***
Anterior corona radiata	0.354	0.354	0.369	0.372
Anterior limb of internal capsule	0.463	0.459	0.470	0.475
**Body of corpus callosum**	**0.497** ^*^	0.514	0.528	**0.528** ^*^
Cerebral peduncle	0.517	0.533	0.530	0.538
**Cingulum**	**0.307** ^*+^	0.331	**0.364** ^**+**^	**0.349** ^*^
Corticospinal tract	0.526	0.531	0.538	0.529
External capsule	0.390	0.397	0.414	0.396
Fornix column body	0.239	0.255	0.327	0.310
**Fornix cres**	**0.370** ^*+#^	**0.426** ^**#**^	**0.440** ^**+**^	**0.427** ^*^
**Genu of corpus callosum**	**0.425** ^*^	0.437	0.464	**0.475** ^*^
**Hippocampal white matter**	**0.293** ^**+**^	**0.304** ^**∧**^	**0.343** ^**+∧**^	0.323
Inferior cerebellar peduncle	0.474	0.477	0.494	0.476
Medial lemniscus	0.526	0.544	0.537	0.525
Middle cerebellar peduncle	0.485	0.490	0.488	0.484
**Pontine crossing tract**	**0.436** ^*^	0.448	0.442	**0.460** ^*^
**Posterior corona radiata**	**0.414** ^*^	0.413	0.437	**0.439** ^*^
Posterior limb of internal capsule	0.518	0.527	0.530	0.534
**Posterior thalamic radiations**	**0.445** ^*+^	0.467	**0.485** ^**+**^	**0.491** ^*^
Retrolenticular internal capsule	0.537	0.535	0.534	0.545
**Sagittal stratum**	**0.416** ^*^	0.431	0.434	**0.444** ^*^
**Splenium of corpus callosum**	**0.548** ^*^	0.561	0.558	**0.575** ^*^
Superior cerebellar peduncle	0.570	0.592	0.566	0.574
Superior corona radiata	0.431	0.429	0.434	0.442
Superior fronto-occipital fasciculus	0.377	0.376	0.396	0.408
Superior longitudinal fasciculus	0.432	0.433	0.449	0.447
**Tapetum**	**0.306** ^*+^	0.327	**0.359** ^**+**^	**0.367** ^*^
Uncinate fasciculus	0.392	0.413	0.426	0.409
**Localized posterior tracts**	**0.430** ^*+^	0.452	**0.467** ^**+**^	**0.478** ^*^

^*^Significant difference between Quartile 1 (lowest visuospatial performance) and 4 (highest visuospatial performance); ^+^significant difference between Quartile 1 and Quartile 3; ^#^significant difference between Quartile 1 and Quartile 2, correcting for multiple comparisons (Bonferroni). **Bold** indicates differences between any visuospatial quartiles. ^∧^significant difference between Quartile 2 and 3.

### White matter integrity and CPC-Q scores

**Figure 2:**
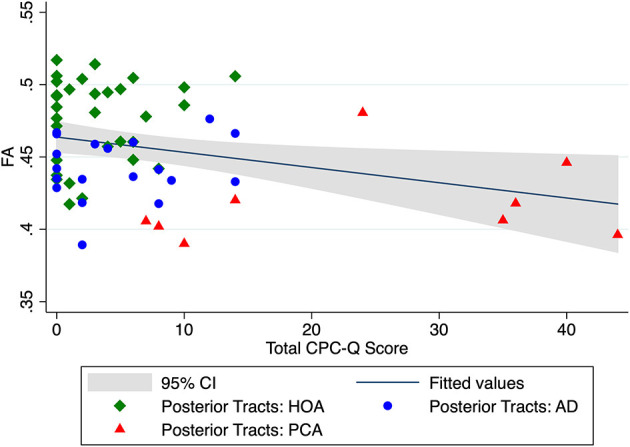
Unadjusted relationship between mean DTI FA of *localized posterior tracts* and total CPC-Q score.

In univariate analyses, the FA of our main *a priori* white matter region of interest, the mean *localized posterior tracts*, significantly correlated with total CPC-Q score (r = −0.31, *p* = 0.018, Figure 2). When controlling for demographic covariates of age and education, the *localized posterior tracts* remained significantly associated with total CPC-Q score [F (3, 55) = 9.56, *p* < 0.001].

Correlations for DTI FA of all white matter region/tracts to both total CPC-Q and visuospatial composite scores are presented in Table 4. Relationships between individual tract/region FA and total CPC-Q score did not survive correction for multiple comparisons; uncorrected values are presented as exploratory results. Two white matter regions chosen *a priori* for the *localized posterior tract* variable were associated with both CPC-Q and visuospatial composite scores: the posterior thalamic radiations and splenium of the corpus callosum. In addition, the body of the corpus callosum and fornix cres were also associated with both scores. The medial lemniscus was associated with the CPC-Q, but not the visuospatial composite. The posterior thalamic radiations were most strongly associated with CPC-Q score (r = −0.34, *p* = 0.009); the corticospinal tracts were the least associated (r = −0.08, *p* = 0.55).

**Table 4:** Exploratory correlational analyses between mean DTI FA for all white matter region/tracts and CPC-Q and visuospatial composite scores.

**White matter region/tract**	**CPC-Q to FA correlation coefficient**	***p*-value (uncorrected)**	**VS comp to FA correlation coefficient**	***p*-value (Bonferroni)**
Anterior corona radiata	−0.148	0.264	0.306	1.000
Anterior limb of internal capsule	−0.103	0.438	0.283	1.000
**Body of corpus callosum**	**−0.323**	**0.013**	**0.484**	**0.044**
Cerebral peduncle	−0.155	0.241	0.350	1.000
Cingulum	−0.218	0.098	0.430	0.285
Corticospinal tract	−0.079	0.550	0.097	1.000
External capsule	−0.169	0.201	0.195	1.000
Fornix column body	−0.126	0.343	0.279	1.000
**Fornix cres**	**−0.330**	**0.011**	**0.502**	**0.023**
Genu of corpus callosum	−0.179	0.174	0.404	0.626
Hippocampal white matter	−0.253	0.053	0.414	0.467
Inferior cerebellar peduncle	−0.145	0.272	0.139	1.000
**Medial lemniscus**	**−0.268**	**0.040**	0.156	1.000
Middle cerebellar peduncle	−0.119	0.371	0.124	1.000
Pontine crossing tract	−0.204	0.121	0.413	1.000
Posterior corona radiata	−0.232	0.077	0.329	1.000
Posterior limb of internal capsule	−0.240	0.067	0.352	1.000
**Posterior thalamic radiation**	**−0.339**	**0.009**	**0.581**	**0.001**
Retrolenticular part of internal capsule	−0.111	0.401	0.147	1.000
Sagittal stratum	−0.192	0.145	0.375	1.000
**Splenium of corpus callosum**	**−0.270**	**0.039**	**0.491**	**0.035**
Superior cerebellar peduncle	−0.155	0.243	0.170	1.000
Superior corona radiata	−0.166	0.210	0.144	1.000
Superior fronto-occipital fasciculus	0.002	0.991	0.258	1.000
Superior longitudinal fasciculus	−0.122	0.358	0.248	1.000
Tapetum	−0.215	0.103	0.426	0.329
Uncinate fasciculus	−0.187	0.156	0.302	1.000
**Localized posterior tract**	**−0.307**	**0.018**	**0.565**	**0.001**

ROI, Region of interest; CPC-Q, Colorado Posterior Cortical Questionnaire, FA, fractional anisotropy; VS Comp, visuospatial composite score; **Bold** indicates p <0.05. Higher score on CPC-Q reflects more posterior cortical symptoms; higher score on visuospatial composite reflect better performance. Significance for CPC-Q to FA correlations did not survive correction for multiple comparisons.

## Discussion

Early and accurate detection of posterior cortical symptoms, with distinction from ocular or ophthalmologic symptoms, is a major need in the clinical care and research of cognitive syndromes. The CPC-Q is significantly correlated with visuospatial neuropsychological measures and discriminates PCA from amnestic AD and asymptomatic groups (5), offering a quick self-report scale that could easily be deployed within clinical settings. To support future dissemination, the presented preliminary neuroanatomical analyses further validate the CPC-Q and deepen its clinical and pathophysiological implications. A focus on white matter is supported by previous neuroimaging studies in PCA (7–10). The integrity of our *a priori* white matter region of interest, the averaged *localized posterior tract*, was significantly associated with CPC-Q scores. Furthermore, the FA of this region of interest significantly differed between the symptomatic and asymptomatic groups, as well as between the lower and higher visuospatial performance quartiles. The *localized posterior tract* FA was not significantly different between the PCA and amnestic AD groups, however. In our exploratory comparisons, the FA of the posterior thalamic radiations and the body and splenium of the corpus callosum were significantly different between the PCA and amnestic AD groups.

The FA of the fornix cres and medial lemniscus were also associated with CPC-Q score in uncorrected comparisons for future hypothesis generation; the medial lemniscus finding could reflect vestibular abnormalities that have been reported by people with PCA, including upside down vision or tilt of the vertical (18). The FA of the posterior thalamic radiations, body and splenium of the corpus callosum, and the fornix cres were also significantly correlated with visuospatial composite scores, though the FA of the medial lemniscus was not. These observations may indicate that the CPC-Q is detecting unique visuospatial/perceptual symptoms that traditional neuropsychological testing cannot. The correlation between the FA of any individual white matter region/tract and CPC-Q did not survive correction for multiple comparisons, but future studies will consider these five regions as areas of interest in additional validation of the CPC-Q.

Separate from the analyses focused on relationships between white matter integrity and the CPC-Q, we more broadly explored white matter integrity by diagnostic group. Our PCA group demonstrated lower FA than healthy older adults in 14 of the 27 available white matter region/tracts; the amnestic AD group had lower FA in 10 of these 27 regions compared to the controls. These results support previous findings of more diffuse and severe white matter degradation in PCA compared to other syndromic presentations of AD (10). However, our PCA group had significantly longer symptom duration, despite younger age, and more severe impairment by cognitive test scores. Therefore, the degree of white matter abnormality, measured by comparisons of individual FA values or number of affected region/tracts, may reflect more advanced disease stage rather than cognitive syndrome.

The findings of reduced FA in a variety of posterior cerebral white matter tracts are of interest from two perspectives. First, with respect to PCA, our data add to existing evidence that white matter is significantly affected in this disease (7–10). Longitudinal studies to identify the sequence of neuropathological events in PCA are clearly needed, however, to establish the role of white matter in pathogenesis. Second, our data showing reduced FA in many posterior white matter tracts among the lowest performing visuospatial quartile support the use of the CPC-Q to identify individuals with prominent visuospatial and visuoperceptual impairments, regardless of their diagnosis. These observations suggest that the CPC-Q may prove useful as a clinical tool for the detection of symptoms reflecting disrupted posterior tracts in white matter disorders of diverse etiologies. Such an instrument would be welcomed in clinical settings where the diagnosis of PCA, and other disorders of posterior cortical regions, presents substantial challenges, including in optometry and ophthalmology clinics.

Limitations of this study include a small sample size, including only nine participants with PCA. Though likely more common than currently thought due to limited screening tools and misdiagnoses, PCA is a relatively rare condition overall. Current efforts toward multi-center collaborations, with harmonization of PCA clinical measures and data sharing, will help to confirm and expand our initial pilot findings (19, 20). The small sample size likely affected the power of the CPC-Q to DTI FA comparisons, especially given the non-normality of CPC-Q data; as can be appreciated in Figure 2, most CPC-Q scores cluster in the <20 range. While the significance of the CPC-Q to FA correlations did not survive correction for multiple comparisons in this pilot study, the uncorrected correlation coefficients are hypothesis generating for planned larger studies moving forward. Despite these limitations, our initial results suggest that integration of a self-reported questionnaire with measures of white matter integrity from neuroimaging may provide additional avenues for early detection of posterior cortical dysfunction and underlying neurodegenerative etiologies.

## Data availability statement

The raw data supporting the conclusions of this article will be made available by the authors, without undue reservation.

## Ethics statement

The studies involving human participants were reviewed and approved by Colorado Multiple Institutional Review Board. The patients/participants provided their written informed consent to participate in this study.

## Author contributions

SH, BB, and VP contributed to conception and design of the study. SH, BB, and DL-P organized the database. SH performed the statistical analysis and wrote the first draft of the manuscript. SH, BB, CF, and VP wrote sections of the manuscript. All authors contributed to manuscript revision, read, and approved the submitted version.
